# Invasive pulmonary aspergillosis is associated with adverse clinical outcomes in critically ill patients receiving veno-venous extracorporeal membrane oxygenation

**DOI:** 10.1007/s10096-018-3241-7

**Published:** 2018-04-06

**Authors:** I. Rodriguez-Goncer, S. Thomas, P. Foden, M. D. Richardson, A. Ashworth, J. Barker, C. G. Geraghty, E. G. Muldoon, T. W. Felton

**Affiliations:** 1 Infectious Diseases Department, Wythenshawe Hospital, Manchester University NHS Foundation Trust, Manchester, UK; 2 Microbiology Department, Wythenshawe Hospital, Manchester University NHS Foundation Trust, Manchester, UK; 3 Medical Statistics Department, Wythenshawe Hospital, Manchester University NHS Foundation Trust, Manchester, UK; 4 Mycology Reference Centre, Wythenshawe Hospital, Manchester University NHS Foundation Trust, Manchester, UK; 5 Division of Infection, Immunity and Respiratory Medicine, The University of Manchester, Manchester Academic Health Science Centre, Manchester, UK; 6 Cardiothoracic Critical Care Unit, Wythenshawe Hospital, Manchester University NHS Foundation Trust, Manchester, UK; 7 Manchester Medical School, University of Manchester, Manchester, UK; 8 Infectious Diseases Department, Mater Misericordiae University Hospital, Eccles Street, Dublin 7, D07 R2WY Ireland; 9 Acute Intensive Care Unit, Wythenshawe Hospital, Manchester University NHS Foundation Trust, Southmoor Road, Manchester, M23 9LT UK

**Keywords:** *Aspergillus fumigatus*, ECMO, Galactomannan, Voriconazole, Outcome

## Abstract

To identify the incidence, risk factors and impact on long-term survival of invasive pulmonary aspergillosis (IPA) and *Aspergillus* colonisation in patients receiving vv-extracorporeal membrane oxygenation (ECMO). A retrospective evaluation was performed of patients receiving vv-ECMO at a tertiary hospital in Manchester (UK) between January 2012 and December 2016. Data collected included epidemiological data, microbiological cultures, radiographic findings and outcomes. Cases were classified as proven IPA, putative IPA or *Aspergillus* colonisation according to a validated clinical algorithm. One hundred thirty-four patients were supported with vv-ECMO, median age of 45.5 years (range 16.4–73.4). Ten (7%) patients had putative IPA and nine (7%) had *Aspergillus* colonisation. Half of the patients with putative IPA lacked classical host risk factors for IPA. The median number of days on ECMO prior to *Aspergillus* isolation was 5 days. Immunosuppression and influenza A infection were significantly associated with developing IPA in a logistic regression model. Cox regression model demonstrates a three times greater hazard of death associated with IPA. Overall 6-month mortality rate was 38%. Patients with putative IPA and colonised patients had a 6-month mortality rate of 80 and 11%, respectively. Immunosuppression and influenza A infection are independent risk factors for IPA. IPA, but not *Aspergillus* colonisation, is associated with high long-term mortality in patients supported with vv-ECMO.

## Introduction

Veno-venous extracorporeal membrane oxygenation (vv-ECMO) is an effective treatment to support patients with severe respiratory failure unresponsive to conventional therapies [1]. Its use is increasing but survival to hospital discharge remains between 50 and 60% [2]. Older age, being immunocompromised, longer duration of mechanical ventilation before vv-ECMO, neuromuscular blockade agents, nitric oxide use and increased extra-pulmonary organ failure are associated with worse outcomes [3–5].

Healthcare-associated infections occur in 26–45% of patients on vv-ECMO and are associated with high mortality and increased hospital stay [6–8]. Fungal pathogens are commonly isolated from adults supported with vv-ECMO [9, 10]. *Aspergillus* spp*.* are identified more frequently in this group compared with those in other groups of critically ill patients [7]. *Aspergillus* in the airways of patients supported with ECMO may be associated with poorer outcomes [10–12]. Several major risk factors have been identified for developing invasive pulmonary aspergillosis (IPA) in critically ill patients not receiving vv-ECMO, such as influenza A infection, higher SOFA score and previous broad-spectrum antibiotic therapy [13–15].

The diagnosis of IPA in critically ill patients is challenging. Severe respiratory failure and coagulopathy make invasive sampling techniques such as lung biopsy difficult. Non-invasive diagnostic tests have not been well validated in immunocompetent patients [16]. A simple, clinical diagnostic algorithm for the diagnosis of IPA in critically ill patients has been externally validated in a large, multi-centre cohort and may have utility in patients supported with vv-ECMO [17, 18]. The algorithm incorporates mycological culture and microscopy of broncho-alveolar lavage (BAL) samples from critically ill patients as a tool to discriminate *Aspergillus* colonisation from IPA.

Here, we describe the incidence of *Aspergillus* infection and colonisation in patients supported with vv-ECMO and identify potential risk factors and the effect of *Aspergillus* infection on long-term survival.

## Materials and methods

### Study design and setting

A retrospective, observational study of all critically ill patients who received vv-ECMO support between January 2012 and December 2016 was performed. As the study was a retrospective service evaluation, ethical approval was not required.

### Study definitions

Patients, aged 16 years or older and with potentially reversible severe acute respiratory failure despite optimisation of conservative therapy, were considered for support with vv-ECMO. Patients with positive *Aspergillus* spp. culture from BAL were classified as proven IPA, putative IPA or *Aspergillus* colonisation according to a validated clinical algorithm [17, 18]. A diagnosis of proven IPA requires histopathological evidence of fungal invasion. For a diagnosis of putative IPA, a patient had a positive culture for *Aspergillus* spp. from any lower respiratory tract (LRT) sample, together with meeting three criteria: (1) compatible signs and symptoms, (2) any abnormality on medical imaging and (3) either a positive semi-quantitative *Aspergillus* culture from BAL without bacterial growth and a positive cytological smear showing branching hyphae or any of the host risk factor for IPA according to EORTC/MSG criteria [19]. *Aspergillus* colonisation was considered when ≥ 1 criteria necessary for a diagnosis of putative IPA were not met. Patients without *Aspergillus* spp. in the airways were included in the non-*Aspergillus* group.

### Data collection

#### Clinical data

Clinical data were collected from electronic medical records including demographics (age, sex), underlying medical conditions, indication for vv-ECMO, duration of ventilation prior to vv-ECMO and duration of vv-ECMO support, *Aspergillus* diagnosis, antifungal therapy and clinical outcome.

#### Microbiological and radiological data

Bacterial and fungal culture findings were recorded. Galactomannan (GM) was measured in BAL [20]. All chest computed tomography (CT) was reported by specialised chest radiologists. Chest CT scans were considered to be “suggestive” of IPA if any of the following were seen: (1) lung cavitation, (2) air-crescent sign or (3) dense, well-circumscribed lesion(s) with or without a halo sign.

#### Statistical analysis

Statistical analysis was performed using GraphPad Prism version 7.0 and IBM SPSS Statistics version 22. The Mann-Whitney test was used to compare GM indexes in patients with IPA with those without. A logistic regression model was used to identify risk factors (bacterial pneumonia, influenza A infection, chronic obstructive pulmonary disease and immunosuppression) for acquiring IPA. A multivariate Cox proportional hazards regression model was used to establish factors affecting outcome in patients receiving vv-ECMO (age, days of mechanical ventilation prior to receiving vv-ECMO support, bacterial or viral pneumonia and *Aspergillus* colonisation or IPA). These factors were included as they have previously been shown to affect outcome in patients supported with vv-ECMO [4]. Kaplan-Meier curves were used to compare survival of patients with *Aspergillus* infection, colonised patients and patients without any evidence of *Aspergillus* over time. Statistical significance was defined as *p* < 0.05.

#### Environmental screening

During the study period, environmental air sampling was performed periodically according to local policy and published protocols [21]. Samples were collected from multiple locations within the Cardiothoracic Critical Care Unit. The agar plates were incubated at 30 °C for 5 days.

## Results

### Patients’ demographics and clinical characteristics

A total of 134 patients, 73 (54%) male, were included. The mean age was 44.3 years (range 16.4–73.4) (Table 1). Respiratory infection was the most common indication for receiving vv-ECMO support with 74/134 (55%) patients with bacterial pneumonia, 23/74 (31%) of whom had *Streptococcus pneumoniae* isolated from BAL. Forty (30%) patients were admitted with viral pneumonia of whom 24/40 (60%) had influenza A infection. One patient with *Streptococcus pneumoniae* pneumonia developed putative IPA. The most common underlying respiratory diagnosis was asthma, 18/134 (13%) of patients. No patients had a diagnosis of allergic bronchopulmonary aspergillosis. The median length of stay on mechanical ventilation prior to vv-ECMO was 2 days (range 1–22) and the median duration on vv-ECMO support was 13 days (range 1–57).

**Table 1:** Patient demographics and clinical, microbiological and radiological findings

	Non-*Aspergillus* group (*n* = 115)	Putative IPA (*n* = 10)	*Aspergillus* colonisation (*n* = 9)
Age, years [median, (range)]	45.4 (16.4–73.4)	51.2 (23.9–64.6)	35.3 (17.8–46.8)
Male [*n* (%)]	60 (52)	8 (80)	5 (63)
Time ventilated before vv-ECMO, days [median, (range)]	3 (1–22)	1 (1–7)	1 (1–6)
Duration of vv-ECMO support, days [median, (range)]	13 (1–57)	26 (7–45)	10 (4–29)
Underlying conditions [*n* (%)]			
COPD	7 (6)	1 (10)	1 (13)
Asthma	13 (11)	1 (10)	4 (50)
Diabetes	7 (6)	1 (10)	0
Solid tumour	2 (2)	1 (10)	0
HIV	0	1 (10)	0
Active TB	0	1 (10)	0
Autoimmune disease	3 (3)	1 (10)	0
Solid organ transplant	1 (< 1)	1 (10)	0
Current pregnancy	1 (< 1)	0	0
Smoking	15 (13)	0	2 (25)
Heavy alcohol consumption	10 (9)	0	1 (13)
IVDU	3 (3)	0	0
Immunosuppression^a^	15 (13)	5 (50)	1 (11)
Others	37 (32)	2 (20)	1 (11)
EORTC host factors [*n* (%)]		4 (40)	0
Prolonged steroid therapy		3 (30)	0
Chemotherapy		1 (10)	0
Indication for ECMO [*n* (%)]			
Bacterial pneumonia	68 (59)	5 (50)	1 (11)
*Streptococcus pneumoniae*	22 (19)	1 (10)	0
Viral pneumonia	31 (27)	5 (50)	4 (44%)
Influenza A	18 (16)	5 (50)	1 (13)
Asthma	7 (6)	1 (10)	2 (22)
Burns	5 (4)	0	0
Eosinophilic pneumonia	4 (4)	0	0
Other	0	0	2 (22)
Isolated species [*n* (%)]			
*Aspergillus fumigatus*	0	10 (100)	9 (100)
Laboratory findings			
BAL GM, OD index > 0.5 [*n* (%)]	21/53 (39)	6/8 (75)	4/8 (50)
BAL GM, OD index [median, (range)]	0.34 (0.03–9.30)	7.4 (0.53–10.08)	0.54 (0.04–9.44)
Abnormal radiological findings			
Non-specific chest CT scan findings [*n* (%)]	106 (92)	6 (60)	8 (100)
“Suggestive” chest CT scan findings of IPA [*n* (%)]	9 (8)	4 (40)	0

Data are numbers or proportion (%)

*IPA* invasive pulmonary aspergillosis, *ECMO* extracorporeal membrane oxygenation, *COPD* chronic obstructive pulmonary disease, *HIV* human immunodeficiency virus, *TB* tuberculosis, *IVDU* intravenous drug user, *EORTC* European Organisation for Research and Treatment of Cancer, *BAL* broncho-alveolar lavage, *GM* galactomannan, *OD* optical density, *CT* computed tomography

^a^Includes patients who are neutropenic or receiving corticosteroids or chemotherapy

Ten (7%) patients had putative IPA and nine (7%) patients had *Aspergillus* colonisation. Six of ten (60%) patients with putative IPA fulfilled the diagnostic criteria within the first 2 days of receiving vv-ECMO support whilst the median number of days supported with vv-ECMO prior to *Aspergillus* isolation was 5 days (range 0–27). One hundred fifteen (86%) patients never had a positive sputum or BAL culture for *Aspergillus* spp. Four of ten (40%) patients with putative IPA had “classical” diagnostic host criteria for IPA according to EORTC criteria [19].

### Risk factors for acquiring IPA and patient outcomes

**Fig. 1:**
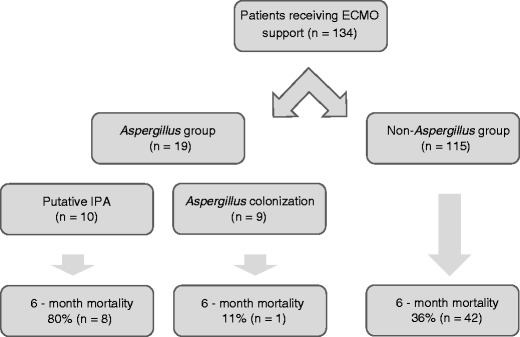
Flowchart and mortality in all groups of patients included in the study

**Fig. 2:**
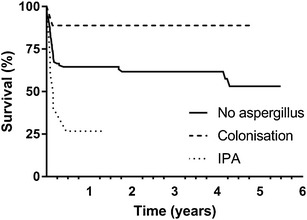
Kaplan-Meier plots showing long-term outcome of patients who received vv-ECMO with putative IPA and *Aspergillus* colonisation and patients with no evidence of *Aspergillus* infection during their admission

Influenza A infection (HR 11.4, 95% CI 1.97–65.86; *p* = 0.007) and immunosuppression (HR 10.75, 95% CI 2.23–51.72; *p* = 0.003) were associated with an increased risk of patients developing IPA (Table 2). The overall 6-month all-cause mortality of all patients who were supported with vv-ECMO during the study period was 38% (51/134). Eight of ten (80%) patients with putative IPA and 1/9 (11%) with *Aspergillus* colonisation had died at 6 months (Fig. 1). Cox regression analysis found mortality was over three times higher in patients with putative IPA compared with that of patients with either colonisation or patients with no evidence of *Aspergillus* spp. (*p* = 0.036) (Fig. 2, Table 3). Additionally, both age and length of mechanical ventilation prior to vv-ECMO were also found to be associated with an increased risk of death. *Aspergillus* colonisation during vv-ECMO, bacterial pneumonia or viral pneumonia was not associated with an increased hazard ratio in the Cox regression model.

**Table 2:** Risk factors for acquiring IPA in critically ill patients receiving vv-ECMO

Risk factor	Hazard ratio (95% CI)	*p* value
Bacterial pneumonia	1.51 (0.30–7.60)	0.615
Influenza A	11.40 (1.97–65.86)	0.007
Chronic obstructive pulmonary diseases	4.83 (0.351–66.49)	0.239
Immunosuppression^a^	10.75 (2.23–51.72)	0.003

^a^Immunosuppression includes patients who are neutropenic or receiving corticosteroids or chemotherapy

**Table 3:** Effect of risk factors on survival of critically ill patients receiving vv-ECMO

Risk factor	Hazard ratio (95% CI)	*p* value
Viral pneumonia	0.49 (0.17–1.42)	0.188
Bacterial pneumonia	0.63 (0.317–1.27)	0.199
Age		0.003
18–49 years	1 (reference category)	
50–59 years	3.44 (1.42–8.33)	0.006
≥ 60 years	3.85 (1.55–9.56)	0.004
*Aspergillus* diagnosis		
Non-*Aspergillus* infection	1 (reference category)	0.067
*Aspergillus* colonisation	0.43 (0.06–3.32)	0.421
Putative IPA	3.31 (1.08–10.14)	0.036
Time on mechanical ventilation prior to vv-ECMO		
< 48 h	1 (reference category)	0.028
48 h to 7 days	2.02 (0.91–4.54)	0.085
> 7 days	3.70 (1.37–9.98)	0.010

### Laboratory and radiological findings

**Fig. 3:**
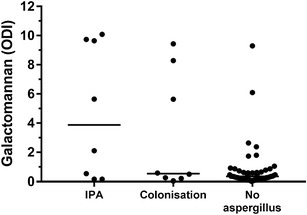
BAL GM mean optical indexes (ODI) measured in patients with putative IPA and *Aspergillus* colonisation and patients with no evidence of *Aspergillus* in their airways

From those patients who had a positive BAL *Aspergillus* culture, all had *Aspergillus fumigatus* identified. GM was measured in the BAL fluid from 53/115 (46%) patients in the non-*Aspergillus* group, 8/10 (80%) patient with putative IPA and 8/9 (88%) patients with *Aspergillus* colonisation (Table 1). Patients with putative IPA had significantly higher BAL GM OD indexes compared to patients without putative IPA

(Mann-Whitney test *p* = 0.029) (Fig. 3). Using a GM index threshold of ≥ 0.5 for a diagnosis of putative IPA, the sensitivity, specificity and positive and negative predictive values of the BAL GM assay were 75, 59, 19 and 95% respectively.

All patients with *Aspergillus* in BAL had a chest CT scan performed. Four out of ten (40%) patients with putative IPA had typical radiology changes suggestive of IPA; 1/4 (25%) had a classical host risk factor.

### Antifungal therapy

Seven of ten (70%) patients with putative IPA were treated with a combination of micafungin and voriconazole, 2/10 (20%) patients received voriconazole and 1/10 (10%) received liposomal amphotericin B. Seven of the nine (77%) patients colonised with *Aspergillus* and 51 of 115 (44%) patients from the non-*Aspergillus* group received antifungal therapy either empirically or to treat candidaemia.

### Environmental screening

A total of 46 air samples were collected over eight separate occasions. *A. fumigatus* was isolated on three occasions (range 1–3 colonies forming units (CFU) per cubic metre of air) whilst *A. niger* was isolated on one occasion. Throughout the study period, the level of *A. fumigatus* in the adjacent outside air was in the range of 2 to 23 CFU per cubic metre of air.

## Discussion

During a 5-year period, we found an incidence of putative IPA and *Aspergillus* colonisation in critically ill patients receiving vv-ECMO support of 7% each. The combined incidence (14%) of *Aspergillus* disease in patients receiving vv-ECMO support is higher than that reported in critically ill adults, which is typically between 0.05 and 7.5% [7, 12, 22, 23]. Many of these trials did not differentiate between *Aspergillus* infection and colonisation and include patients receiving both vv-ECMO and veno-arterial ECMO as a combined category [7, 24], making true comparison difficult. Aubron et al. [11] described 11 patients from 151 receiving ECMO support with *Aspergillus* disease but just 2 (18%) patients were supported with vv-ECMO. Hospital-acquired *Aspergillus* infection is very unlikely as environmental screening demonstrated an acceptable number of environmental *Aspergillus* conidia with no evidence of excessive fungal contamination. Our study has identified immunosuppression and influenza A infection as independent risk factors for IPA. Thus, the high incidence of *Aspergillus* infection in our patients may be related to the high rate of patients with influenza A.

IPA, but not *Aspergillus* colonisation, was associated with a significantly higher mortality at 6 months in patients receiving vv-ECMO (80 vs 11%). This difference in outcomes between infection and colonisation was also previously described by Contou et al. in critically ill patients with acute respiratory distress syndrome (ARDS) [25]. The multivariate Cox regression model, which incorporated risk factors known to be associated with excess mortality in patients receiving vv-ECMO, demonstrated a three times greater hazard of death in patients with putative IPA compared to non-*Aspergillus* group. Previous studies on critically ill patients, which include immunocompromised patients, have shown mortality rates due to IPA of over 75% [11, 14, 22]. In comparison to previous studies, which only focused on hospital mortality, we have provided long-term follow-up data which shows that patients die early in their critical illness from IPA.

Diagnosing *Aspergillus* infection in critically ill patients remains challenging. We found that 60% of patients with putative IPA had non-specific changes of IPA on the CT scan. Higher levels of GM in BAL were observed in patients with putative IPA than in colonised patients or patients with no evidence of *Aspergillus* in their airways. A GM in BAL with an OD index ≥ 0.5 showed a negative predictive value of 95% suggesting that this test may be useful as a rule-out test for IPA in this patient population.

The most appropriate antifungal therapy for IPA on vv-ECMO is still unclear. In the present study, the number of patients with *Aspergillus* infection and colonisation was too small to find significant differences with different treatment regimes. The role of antifungal prophylaxis is still unclear but it may be beneficial in high-risk patients supported on vv-ECMO (e.g. influenza infection, age over 60 years and prolonged previous mechanical ventilation). This strategy has been previously used in high-risk immunocompromised patients [26] but further studies are needed to specify its role in patients with vv-ECMO support.

This study has some limitations mostly related with the retrospective analysis of the data at a single centre. The relatively small number of putative IPA and colonised patients indicates that these results of both regression models should be viewed as exploratory rather than confirmatory. Severity of illness score was not recorded on the medical records of patients who received vv-ECMO support. GM was collected in only 69/134 (51%) of patients, so care is required interpreting sensitivity and specificity analysis. Larger prospective studies are required to identify clinically important biomarkers of IPA and optimal antifungal regimens.

In summary, *Aspergillus* infection in patients undergoing vv-ECMO support is associated with poor long-term outcome. Prolonged mechanical ventilation and older age were associated with worse outcome. Previous influenza infection was identified as a risk factor for developing IPA. GM in BAL may have a role as a rule-out test for IPA in this population. Optimal antifungal therapy remains unclear and further studies are required to optimise diagnosis and management in patients receiving vv-ECMO.
